# New Methods of Treatment in Phthisis. I.—The Roussel Treatment

**Published:** 1891-10-03

**Authors:** 


### The Practitioner's Mirror and Retrospect.

*[The Editor will be glad to receive offers of co-operation and contributions from members of the profession. All letters should be addressed to* The Editor, The Lodge, Porchester Square, London, W.]

### MEDICINE.

### NEW METHODS OF TREATMENT IN PHTHISIS.

#### I.—The Roussel Treatment.

Whether we believe or not in any new or any old method of treating disease, it is certainly our duty to pause and consider the claims of all therapeutic methods which appear to be based on good and honest clinical work, and sometimes too we have to consider methods that have not even this recommendation. Now, it was only this time last year, the medical world, that is to say the great body of the medical profession in the most enlightened portions of the world, went mad over the Koch treatment, and now forsooth the very men who were loudest in their unstinted praise of a half-understood, and less than half-tried method, condemn it as beneath notice. Perhaps it is well then that the Koch treatment should have become relatively old and out of date, and has been replaced by about six freshly advocated methods, for it will at least allow one of our most careful, scientific, and painstaking investigators the medical world has ever possessed, to pursue his uncompleted investigations until they are followed by some more complete and worthy result.

It is well then to note the nature and claims of these new methods, for if we discredit any of them, it is desirable to know what it is we disbelieve in, and, at any rate, it is probable that we shall derive some grains of gold from these quicksands on which fair reputations are so often lost.

Dr. Roussel's method appears firstly to have the great advantage of being harmless if properly carried out, and further, it seems to give encouraging results. He starts with the theory that the symptoms of phthisis are due to an effort on the part of nature to throw off the microbe which is killing the organism, that by suppuration and expectoration the germ is thrown off, that the raised temperature destroys these chemical products by burning the leucomaines which are circulating in the blood; the abundant night sweats, the diarrhœa, and surcharged urines are all so many methods of elimination, and are the defensive methods nature employs against the invasion of the tubercle bacillus. He argues, therefore, that our treatment should aim at attacking the microbe, and that we ought not to try to diminish the power of those weapons which the body employs in self-defence. New remedies taken by the mouth injure and disturb the digestion, thereby weakening the patient, while they altogether fail to reach the lungs in sufficient strength to successfully attack and overcome the materies morbi in the lung where the germs are situated. Firstly, Dr. Roussel lays stress on general hygienic treatment, good ventilation, sunlight, and warm clothing. With these alone a certain amount of improvement generally follows, and thus it is good hygienic surroundings rather than the drugs as usually employed, which have the beneficial effect on patients so treated; and to prove that it is due to the efficacy of a drug that the improvement takes place in a patient, the improvement should take place when the environment remains unaltered. The plan recommended by Dr. Roussel is to inject various antiseptics, of which eucalyptol and thymol seem to be his favourites, under the skin, whence they become rapidly absorbed and are carried direct to the lungs. Eucalyptol is employed in a twenty per cent. solution in pure sterilised olive oil; this being a substance not foreign to the usual nutrition of the body, it is easily saponified and absorbed by the organism. The medium by which the medicaments are introduced is a matter of extreme importance; thus, mineral oils, such as liquid vaseline, he carefully avoids as these substances are foreign to the human body and are liable to cause irritation and to remain unabsorbed. Very soon after the injection the odour of the volatile oil is noticeable in the breath of the patient, and subsequently both the urine and the sweat acquire the same odour. The ordinary hypodermic needle is unsuited to these injections, being difficult to keep aseptic. The doctor uses a special celluloid syringe, which is transparent and easily sterilised. The needles are of steel covered with platinum, and are made long so that the injection can be inserted deep under the skin and far from the point of puncture. Of course, the treatment must be continued over a considerable period as the volatile oil must permeate the whole body, and thus the body must be, so to speak, embalmed. Dr. Roussel has employed this method since 1883. A female patient, the first case, was sent to Dr. Roussel by Dr. Fauvel. Her right lung was diseased, and numerous bacilli were found in the sputum. After twelve months' treatment the bacilli and all the graver symptoms had disappeared; she gained weight, and has ever since been able to work. So great was his success that Dr. Roussel felt justified in bringing the matter before the Society of Practical Medicine in 1888, at which time he showed the society eighteen patients, all of whom were suffering from phthisis in various stages. After twelve months' treatment he was able to bring sixteen of these before the same society. Nearly five thousand injections had been given and yet there was no sign of any inflammation having occurred as the result of the injections. On the other hand, the patients were all greatly improved in general health and some apparently cured. In several cases examination of the sputa showed a complete absence of tubercle bacilli. In 1886, Dr. Ball, who had been carrying out Dr. Roussel's method at the Laennec Hospital, issued a report on twenty-one cases. Of these, ten were able to leave the Hospital for Incurables and resume their work, five were still under treatment at the time of the report, and six had died. Dr. Ball found that the injections had a very marked effect on the septic symptoms of phthisis, *e.g.*, night sweats, diarrhœa, etc., there was a reduction of the amount of expectoration, of the temperature, and an improvement in the appetite. But Dr. Ball did not claim that these few cases were sufficient to justify the assertion that a cure for phthisis had been found, although they gave great encouragement.

These are the general outlines of Dr. Roussel's method, and it will be observed that it differs from that already employed by many physicians in attention to detail, and in his having overcome many of the greatest obstacles to its successful employment, firstly by improving the instrument employed, and, secondly, by the vehicle by which the active ingredient is carried into the body.*

In connection with this method of treating phthisis by these oils, we may refer to the researches of Dr. Omelchenko, (*Lancet*, April 4th, 1891) on the action of the vapour of ethereal oils on bacteria. Cultures of the bacilli of typhoid fever, phthisis, and anthrax were severally exposed to a continuous stream of air saturated with the vapour of certain ethereal oils, and the time it took to destroy the bacilli in each case was carefully observed. It was found that if the air was not quite saturated the effect was very markedly diminished. The bactericidal properties of the different oils were found to be greatest in oil of cinnamon, after which the others may be arranged with reference to their bactericidal power in the following descending order: Oleum fœniculi, oleum lavendulæ, oleum caryophyllarum, oleum thymi,

* *Lond. Med. Rec.*, Jan. 20, 1891; *Lancet*, Nov. 15, 1890.

oleum menthæ piperitæ, oleum anisi, oleum menthæ crispæ, oleum eucalypti globuli, oleum camphoræ japonicæ, oleum valerianæ, eucalyptol, French oil of turpentine. The oils of lemon and of roses had an exceedingly slight action. From these results it would appear that Dr. Roussel might possibly have employed other oils than eucalyptus and thymol with greater advantage. On the other hand, however, Dr. Omelchenko found that when he passed the air through emulsions instead of through the oils themselves the amount taken up was materially less than in the latter case, and so it would appear that the bactericidal capacity of any one of these substances depends largely upon its volatility.

